# Physiological Researches

**Published:** 1838

**Authors:** M. Gerdy

**Affiliations:** Professor of surgical pathology, in the Paris faculty of medicine, and senior professor of physiology


### Art. IV.—Physiology of our Sensations.

*Physiological Researches on the Sensations, by M. Gerdy,* professor of surgical pathology, in the Paris faculty of medicine, and senior professor of physiology.

[Translated from the *Archives Generales de Medicine,* for the Western Journal.]

Our sensations are so varied and so imperfectly analysed, that they form almost a new topic, richly deserving attention, both physiologically and pathologically. The study will enable us to distinguish a multitude of sensations heretofore confounded with one another, to know them better and to avoid that confusion which should no longer appear in science.

Physiologists understand sensation to be an impression received, transmitted and perceived, and such is a natural idea; I think, on the contrary, that we generally understand sensation, an excitation of which we are conscious, and which we refer to the organ excited. Thus we define it, *an excitation felt in the disturbed organ,* although perception is accomplished in the brain.

The most learned or the most ignorant would say, I have felt it, I have touched it with my finger; my hand is sensible, the skin is a very sensitive part. In speaking thus, do we not say that sensation resides in the hand? When we perceive a sensation, as burning the finger, there is an impression received, the sensation is transmitted to the brain by means of a nerve, finally the brain is excited and *perceives* or is conscious of the sensation. May we not designate this complex phenomenon by the name of *sensorial perception?*

If through ignorance it were generally believed, that perception resided in the sensitive parts, would the name of sensation be applied to the phenomena which follow in sensorial perception? Undoubtedly it would. Sensation applies to what passes in the organ disturbed, for the sensorial transmission through the nerves is not felt nor generally known. Hence the parts that experience sensation are styled *sensitive*, and hence physiologists themselves are constantly designating as sensations, the first act of the complex phenomenon of sensorial perception. Do they not say that the brain *perceives* the sensations received by the organs? Now this is admitting that sensation is distinct from, and that it is completed before perception; Limitation which is indeed the fact, for the brain perceives without feeling. The most ignorant clown knows that he feels by means of his skin, tastes with his tongue, hears with his ears, sees with his eyes, &c., and he well knows that he does not experience these sensations within his skull.

If we understood the exact meaning of the words *sense*, *sensation*, *sensitive*, we should not say that the brain was the seat of sensation and at the same time that it was not sensitive. In restricting the meaning of the word sensation, I have rendered the language of science more precise and more logical.

To avoid all doubt and error in this matter I inform the reader, that the words sensation or impression, which are nearly synonymous, will be constantly employed to express the *change* or phenomenon existing in an excited organ. Sensation and perception cannot be synonymous, for there are perceptions that do not arrive through the medium of sense—thus a reminiscense is the perception of anterior sensation, but not an actual sensation. The consciousness of an emotion that we experience, or of an idea passing through the mind, is not a sensorial perception: a sensation then is not a perception. A contrary mistake caused Condillac, Destutt de Tracy, and their followers, to commit many errors while playing perpetually on the word sensation. I am sorry to see so superior a mind as the illustrious Gall, falling into the same error. I shall never apply the word to the transmission and perception which follow, for every body, even the physiologists, apply the word *sensitive* solely to the organ which feels; because they cannot apply it to the nerve nor to the brain, neither of which are sentient. Finally, to avoid all obscurity, I shall recognize sensation only in the sensitive organs; and pass to the analysis of the sensations.

*Different modes and species of sensations.*

They may be perceived or unperceived. 1st, Among the first, there are those which we call physical because of their origin; 2d, others consist of sensation of activity of the organs and are impressions of *activity;* 3d, others caused by weariness, the sensations of *fatigue;* 4th, others arising during the rest of the organs, which are *physical wants;* 5th, others arising from unknown causes, and appear to be spontaneous or morbid sensations.

All these kinds or different modes of perceived sensation, comprise other species. But as attention and habit modify considerably the ideas derived from sensations, we must add two other forms of impressions to the five just mentioned, to complete the table.

*Sensations that are not perceived.*—These embrace the excitation which causes the contraction of an involuntary muscle, of a voluntary muscle in a limb that has been amputated, or in an animal just dead—also the impression made by fire upon a limb in which the nerves have been tied or compressed high up; finally, as more analogous to sensation than to any other phenomenon, the effects of a contagious virus, and the impressions of medicines introduced within our organs, whose consecutive effects alone we recognize.

When the fibres of the heart, or intestines are touched when they are irritated by pricking or tearing, &c., although the animal give no sign of suffering, still the contractions of the heart are quickened, those of the intestines are roused if dormant, or they become more palpable. Even after amputation when we irritate the fibres of the part the muscles sometimes contract so actively that the fingers are manifestly flexed. The same experiment with a muscle that has been removed from the body or with an animal that has been knocked down, or decapitated, or with electric sparks, even twelve hours after death, produces the same effects, both on the muscles of animal and of organic life. Mr. Andrew Ure, thus excited horrible movements in the head of a criminal that had been severed from its trunk.

In all these cases, since the muscles contracted under the influence of excitants, they must have received an impression, they must have felt. None of these movements were communicated or transmitted, all are evidently the result of an excitation which aroused in these organs a slumbering but not extinct faculty.

When pathologists speak of paralysis of sensibility in apoplexy, we must not interpret the expression literally. Similar phenomena are observed when the nerve that conveys sensation is cut, the part and the nerve remain apparently insensible, but when cicatrized, do not the limb and the nerve become sensitive when irritated ? Will it be supposed that the nerve was really insensible below the section, or that the cutting a nerve can destroy its sensibility ? Is it not clear that the cicatrization of a nerve can only reproduce its conducting power, which it had lost by being cut, though it experienced sensations as before ? Is it not evident that cicatrization can no more give new powers to the nerves below than cutting could destroy them ? Let us suppose the contrary, and we decide that the nerves and their terminal divisions do not in themselves or from their organization possess the faculty of feeling, but that it is derived from above, is expanded there in the healthy state, and withdraws or vanishes when the ligature destroys the continuity, this would be absurd. It is the same with the contractility of the voluntary muscles, the section of their nerves does not paralyse their contractility, as may be proved by irritating the cut nerves and thereby producing contractions; but the section of these nerves prevents the voluntary excitation coming to the muscles, which remain paralysed from the will without being really so, as the skin that has had its nerves cut appears insensible though nothing proves it to be really so.

Another example of inappreciable sensation is found in the writer, actively engaged in composing, who does not perceive the cold until his benumbed fingers refuse to hold the pen. When a disease is contracted by immediate contact, we are unconscious of any sensation, still, sooner or later an organic lesion is developed in the point exposed to contagion, vital movements, which are not transmitted mechanically—if they result from a particular excitation, the affected point *has felt it.* When mercurials excite salivation, purgatives and emetics, the biliary secretion, sudorifics, perspiration, &c., without our being conscious, it is plain that they have produced upon the salivary glands, the liver, or skin, an impression of some kind, which though unperceived has nevertheless excited and increased their action.

Since in all the cases we have analysed, there has been an excitement and consequently local impressions, not perceived but attested by movements which they have provoked, must we not conclude that there are in the economy, latent and local sensations? I will only introduce one order of facts. Fauvel and Méry have each shown a child born without brain or spinal marrow, that lived several hours, and gave evidence of sensation, one survived twenty-one hours, and even took nourishment, and therefore felt the want of the food that was offered, for he took it. Who will venture to say that he was conscious of these sensations?

If any one objects to my calling these phenomena unperceived sensations; if for want of restriction the connection with sensations be not perceived, and if it be not known where to refer them, let them be called by some other name, and not left out of works of science as has happened heretofore; making it apparent that we could not distinguish the facts connected with physiology, and that our views are too contracted to embrace their whole extent.

These unperceived sensations are so obscure and so little known that we shall not further discuss them, and the generalizations which follow, may be equally applied to them, as to the sensations which we perceive.

1st. The *physical sensations* are either general or special. The general physical sensations are produced by physical or chemical agents; heat, electric strokes, weight, motion, chemical combinations &c. These sensations are not all observed, nor in the same degree in every tissue, nor in every healthy or morbid condition of the frame. They present remarkable though unobserved characters. They only convey the idea of an impress, of the presence of an exciting agent, or of pain, but do not exactly acquaint us with this agent—thus whether a recent or inflamed wound or an exposed nerve be touched with the instrument, the patient recognizes the contact of the probe without distinguishing its form; if it be burned or pinched, or cut, he commonly suffers, but can illy distinguish the sensations experienced, and will refer a burn to a pinch, or the latter to the knife, attributing to one action that which belongs to another. I beg the reader to dwell upon these truths, which will prove very important as we proceed —in teaching us the facts which belong to general physical sensation, and to distinguish it from special physical sensations, from that of touch for instance, which teaches us the form, surface and size of bodies, their dryness, or dampness, &c., a sensation that has been confounded with general physical sensibility.

*Especial physical sensations* are impressions transmitted by a definite agent, upon one or more specified organs:—such are sight, hearing, smell, taste and I would even add those sensations that have been neglected by authors different from the five senses, as tickling, the effect of ammoniacal vapours upon the conjunctiva, which have been confounded with those of general sensibility or with touch. They are distinguished from the first because they are peculiar to the parts wherein they are experienced, and from the latter, because they do not convey any idea of consistence, form, or other qualities of bodies. To this class I would also refer the perceived impressions that are generally caused by medicines, which, for want of a better name, we may call, *medicinal sensations*, in reference to the action of drugs of which we are conscious.

They vary according to the seat, intensity of the impression they make upon us, the effects which follow, the condition they require, and the influences which modify them.

Medications which produce peculiar sensations independent of taste, are narcotics and especially opiates, alcoholic drinks, strychnine, cantharadine &c. Thus light doses of opiates produce a peculiar dullness of the organ of thought, and create a desire for sleep, and a general obtundity which is not destitute of charms. Alcoholic drinks produce analogous effects, causing the jollity of drunkenness. On the contrary, the sensations caused by strychnine are acutely painful, causing animals to utter piercing cries. The other sensations of tact, different from touch properly so called, are distributed among many organs, and especially the skin and the mucous membranes near the natural openings—their discussion will be resumed when we come to analyse the sensations of the skin, mouth, nose &c.

Physical sensibility is not a unique faculty, as might be supposed from reading works of physiology, but is much more extended and varied than would be supposed, and comprises several species, distinct and independent of each other. There are facts of this kind which have obliged me to recognize the *multiplicity* of vital properties a principle, true as fruitful, and as a theory, large as that of Bichat, is contracted.

2nd. *Sensations of organic activity*, constantly give us consciousness of life, so evident, that it is surprising authors do not speak of it, they are readily perceived in the contraction of our muscles, contributing to our ideas of distance and space. It is not, as Buffon said, speaking of sight and the other senses, it is not touch which teaches us the distance of objects when we are obliged to transport ourselves from one place to another, it is the sensation of the activity of the organs, and the more or less extended view of objects successively passed.

We feel perfectly well, acts and emotions of the understanding, so well that we can study them by observation, distinguish them by analysis, judge them and know them. In this way we now penetrate some of the mysteries of intellect. We equally feel the activity of the chest, the larynx and the organs of speech in the production of the voice and pronunciation; and the activity of some of the digestive organs during digestion. We may feel ourselves breathe, but we do not habitually feel the action of the lungs, nor the heart, and never that of the vessels, glands nor lungs during circulation, secretion, nutrition or calorification.

3rd. *The sensations of fatigue* arise from the excessive action of the organs, whether in the intensity or duration of their exercise. They are distinguished from the preceding in persisting after the activity of the organs, they are only perceived in those organs which are active and at rest by turns, and not in those which like the heart and lungs, are never really at rest. When slight they are bearable, when intense, they are so painful as to demand inaction or to render the organs powerless. Fatigue is the limit which nature seems to have placed upon the activity of animals, and which the most indomitable courage cannot overcome. Rest composes the exhausted organs, repairs or renews their spent powers, and reanimates, as it were, their energies which were nearly extinguished.

4th. *Physical wants*, so called in opposition to moral andi ntel-lectual wants, are those of breathing, drinking, eating, &c.—These sensations arise during repose of the organs and are thus opposed to the preceding, which arise from excessive activity.—While we are equally fatigued by repose, and thereby forced to act, as by exercise and obliged to rest, we pass alternately to these different and contrary wants, oscillating continually between two opposite states of activity and repose. All these wants are either natural or artificial, or sometimes both.

The *natural wants* are spontaneously developed in all men, they are those of feeling, thinking, moving, eating, breathing, &c.—The *want* to feel, to think, to act, is the cause of our activity or activity itself. It is evident that the want to feel is not manifest in senses at rest, and that their activity appears essentially subject to that of the understanding; but may it not be said when we have an itching, that we experience the want of a contrary sensation, is it not to drive away this feeling and supply the want, that we sometimes scratch ourselves till the blood flows, with evident gratification? We may even say that the desire to move, to take nourishment, &c., are in the abstract wants of sensation.—As the understanding acts independently of excitants or influences of nature, the intellect never perceives the least want, without acting it out irresistibly, in the darkness of the night as in mid day, but this work is insufficient for persons long accustomed to more fatiguing intellectual labor.

The desire of action on the part of the muscles, is manifest; the yawning and stretching when we wake, the matinal crowing and flapping of the cock, the roaring of the lion, while he opens his mouth, stretches his limbs, and beats the air with his tail, are so many evidences of the fact that no farther proof is required.

Hunger and thirst, the desire to breathe, are so evident sensations, so imperious and so well known wants, that it is unnecessary to attempt a proof of their reality.

To satisfy these wants in moderation, is a source of pleasure and health, but to resist them is painful and sometimes even endangers life. It is admirable that the most important, pressing and imperious of all our wants that of respiration, has not been abandoned to the neglect or caprice of our will, but conducted by instinctive and involuntary acts.

The *artificial wants*, as those of smoking, the desire of alcoholic drinks, &c. are, when developed, as imperious and tyranical as the natural, they torment us with inquietude, ennui and insupportable melancholy, and if not satisfied, disturb the health, if gratified, they cause great pleasure, awaken the intellect and inervation, and through this medium, a host of languishing functions, and bestow upon the whole economy, new powers, freedom and activity.

The natural wants may become in part artificial by the influence of habit; thus an active and laborious life, in which the mind or body has been severely exercised, renders idleness so painful as to endanger the health.

5th. The *spontaneous sensations*, are generally morbid actions, although they may present themselves in a healthy state.—Their permanence constitutes disease by the pain they cause and by the disturbance they leave behind them. They are of several kinds, itchings, pricking, creepings, chills, flushes, pains, and some peculiar morbid sensations, as the *aura epileptica*, *bolus hystericus*, and drumming in the ears, cerebral conjestions, &c.

*Should we attribute these spontaneous sensations often without visible alteration, to an inappreciable, but real organic lesion?* I think that it would be good logic, without transcending the facts furnished by observation, to ascribe them to an alteration of the sensibility. But, it will be said by those who trace every thing to anatomical alteration, how can we admit a lesion of function and consequently a change of the phenomena, without a preliminary organic lesion? Let us admit for a moment that there can be no derangement in a vital function, such as sensibility, or in the sensations derived from it, without some preliminary organic lesion; but this itself necessarily results from a lesion of the nutritive faculties or functions. Well, if we are forced at last to admit a first lesion of the faculties which preside over the molecular changes of our tissues, why not admit that one other property, sensibility may be thus primarially affected? It were indeed closing the eyes to the light, to reject that which is proved every day: what is it indeed but sensibility, that becomes exalted, dulled, and modified in a thousand ways by exercise, under the influence of the slightest excitant; or an imperceptible breath that glances upon the surface of the skin? It will be said, this breath has produced a change in the molecular state of the skin, which caused sensation—for it is impossible to admit that the skin, which feels nothing actually, should feel without having changed its material state, since there is no effect without a cause. This reason will be so continued and carried out that it will be impossible to escape. I will first observe that this is asserting, not proving the question—Our skin is so tough that we cannot tear it to derange the molecules, but we are called upon to admit that the slightest breath may disturb them! Besides when we abandon ourselves to such vague suppositions, will it not appear that the vital nutritive function which holds the particles of the skin together, would first have been altered, without preliminary organic lesion, and consequently that there must always be first a vital lesion? The organic alteration cannot be prior to the vital lesion, since it has been caused by a mechanical or chemical agent, sufficiently powerful to produce instantly an intimate alteration of the tissues. Such theories will only suit superficial minds. Indeed how can a derangement of the molecules in a sentient organ, explain the mystery of the phenomenon of sensation?

If physiologists reasoned and analyzed oftener, they would not have confounded the morbid pains we have mentioned, with physical pains, and consequently the very different sensibility of the two, nor would they have persisted in saying that all the tissues became sensible when inflamed. This language is so deficient in accuracy that it leads directly to error. Thus, to cite a single example, from this operation, one would suppose, that the bones being inflamed, their osseous tissue would be sensitive to mechanical or chemical irritants, which is not the case, but these are spontaneous morbid pains that arise from morbid sensibility, and not from general physical sensibility, for they have none. These are facts which the practice of surgery proves every day in our large hospitals.

*Sensations of attention.* As their name would lead us to suspect, these are complicated with the attention. They are generally acts of the will, reflected or not, and instinctive, which give rise to movements, equally voluntary or involuntary, destined to favor the accomplishing of sensation. They appear more distinct and perfect than the inattentive sensations; but we will show that this is only an illusion.

Walking carelessly along, if we are surprised by a bird passing rapidly before us, we see it; but find it difficult to tell its form or color. If it have awakened our attention and should again flit by us, we look at it, and follow it through space by a voluntary or involuntary movement, and observe accurately what kind of bird it is. If we hear a voice, or low conversation, we instinctively incline the head towards it, and listen, and may thus hear a colloquy that would have escaped us but for the extra attention.

Attention resembles a magnifying glass that enables us to see minute parts of an object; but how does it produce this result? Is it by rendering the sensations more decided? Such is the opinion of many authors and especially of Adelon; he says: "The will arouses the nervous part of the organ of sense and increases its action, as is proved by the greater intensity a sensation always has, when perceived with the will and attention."

If attention were an action belonging to the senses and to them exclusively, the proposition would be logical, because the senses alone would be modified; but, since attention is a state of the mind or brain, which commands and governs sense, it is possible that the change resides in the mind exclusively, and then the proposition may be false. Let us analyze this difficulty.

When we touch or look at any thing with great attention, do we feel that our hand or eyes are more sensitive, that they really receive a more lively and energetic impression than at other times? I have never observed it. But is not the mind more powerful and active, do we not find that it judges with increased ease and rapidity? It cannot be doubted. Then attention does not render the hand more sensitive, bnt the mind more powerful and correct in its decisions. It is very doubtful whether the senses are at all quickened.

We will cite two remarkable examples which will prove that attention brightens perception and judgement only but not sense.

When we see a bird far off and listen to its notes with great attention, if any one pass between us and the bird, we will see him without observing closely; if he speak, we will not understand him. But if our senses be in *erection*, if they be attentive, to use the language of M. Adelon, how does it happen that our eye has not recognized the individual who passed by us, nor our ears distinguished the words he spake, although we both saw and heard him? It is because attention does not render sense more active, and because the sensations are only well observed when the mind is fixed by attention. Perhaps the mental state may increase the sensibility in the sufferings of hypochondria, which seem sharpened by the attention of the patient, but I will not venture to make the assertion.

What has been said of the senses is so true that the vulgar are struck with it, though they cannot account for it. Do we not every day hear people excuse themselves, when spoken to, by saying I did not observe you, I was absent, my mind was occupied. It will not therefore be supposed that they wish to say they did not see nor hear, certainly not—their minds were otherwise engaged. Thus it is, that the inattentive sensations escape unobserved, like the unperceived, or rather the perception of them is so vague that we have no precise idea of the particular agent that produced the impression. The attensive sensations on the contrary, inform us of it with the utmost precision. We shall see further, that exercise and practice add to the certainty and promptitude of our judgment.

Hence there is no difference between the perceptions greater than that which arises from the presence or absence of attention. Every sensation then ought to be successively studied in these two modes. In the case of an attentive sensation, we should always direct the voluntary or instinctive movements, to ascertain the excitant which caused it, and to multiply or enforce the impression.

*Repeated or accustomed sensations.* The repetition of excitants, or the habitual exercise of the senses produces different effects. Sometimes the sensibility will be exalted, at others blunted; in some cases it renders disagreeable, those sensations which ordinarially please, and often renders those sensations agreeable, which were first disliked, and thus creates tyrannic wants.

1st. Reiterated sensations, incapable of wounding the tissues, often irritate and inflame the organs—thus walking produces chafed feet; a strong light inflames the eyes, &c.

2nd. On the contrary, we often see cooks and blacksmiths handling hot coals and iron, that another person could not touch; our artificers work all day without hurting their hands in the least; our foot-soldiers, after a long march, have neither blisters nor chafes on their feet; In both cases there has been at first, more or less irritation, which became blunted by a secondary effect.

3rd. Sensations which were first very pleasant, may become disagreeable and create a decided disgust—this happens with the smell of food that has made us sick, or when we have used one kind of food a long while; in the latter case the taste is not blunt-d but perverted, for the sensation is not that of insipidity, but is actually disagreeable.

4th. Sensations at first disagreeable, often become grateful and their absence a tyrannic want; thus many highly flavored articles of food and luxuries, at first repugnant, soon become delicious. Some, as strong drinks, become necessary, and are irresistible wants. We will observe one very remarkable general fact, the most highly flavored articles only, become very agreable and sometimes necessary; physically as morally, we never have a strong desire for insipid things.

In the face of these various modifications of the sensations or sensibility, what becomes of Bichat's assertion, that *habit blunts the sense and perfects the judgment?* It were well to call it an error, an assertion too much generalized in its first member, for it is only true in one of the four modifications which sensibility experiences from repeated excitation; the second part of the proposition is true, at least it appears so to me. Though M. Adelon attributes this improvement to the senses themselves and not to the understanding. That we may not misinterpret this author, we will cite his words: "If the organ be not sufficiently exercised, on the one hand, it will not be completely developed, nor, on the other, will it acquire in its action, all the quickness and precision of which it is capable, and *grows rusty*, as it were, for want of wear. If, on the contrary it be too much used, it wastes away and breaks." First, I think that the development of sense by exercise is a chimera; it has never been demonstrated that the skin on the fingers of the blind is unusually developed, that the tongue or its membrane were greater in gourmands or cooks; that the nasal cavities or membrane were larger in perfumers; that the eye was larger in the painter, or the ear in musicians, in whom these organs are very much exercised.

Secondly, the senses do not acquire quickness, precision and perfection by exercise, they are not educable. It is the mind which acquires this precision and perfection by exercise and education. Behold the painter whose eyes are enfeebled by age, but who yet sees well enough: do you think that he distinguishes less correctly what falls within the sphere of his vision, than an ignorant person with sharp eyes? Will the musician who hears less distinctly, judge less accurately, the merits of a composition? If you think so, undeceive yourself: They both distinguish and judge by the lights of their intellects if they see and hear, be assured they will judge as well as in their youth, and better, as age will have increased their knowledge and perfected their judgement. This theory of the influence of exercise upon the senses, is connected with that of the same author, on the influence of attention on the sensations; one appears to me no better than the other.

To sum up, there are perceived and unperceived sensations, of the first, there are physical sensations, those of activity, of fatigue, physical wants, spontaneous and morbid, attentive and repeated or habitual sensations. The physical sensations are general or special, the latter are tactile, those of contact, of medicine, taste, smell, sound or light. The sensations of activity are varied as the tissues in which they are found. The same may be said of physical wants. Spontaneous or morbid sensations are still more varied. Attention renders, the sensorial perceptions deeper and more just, but does not give activity to the sensations; finally habit sometimes blunts, sometimes exalts them but never confers an increased delicacy upon the senses themselves.

All these varied modifications, all these species of sensations except those fixed by attention and habit, belong to so many properties of feeling distinct from one another. There are then a multitude of various sensibilities in the animal economy, instead of one, as we might be led to believe by our physiologists, who refer all the sensations to an unique principle: *sensibility*, or at farthest to a second, illy founded property: the *organic sensibility* of Bichat.

